# Optimization of vacuum frying condition for producing silver carp surimi chips

**DOI:** 10.1002/fsn3.1077

**Published:** 2019-07-03

**Authors:** Jiamiao Hu, Hongliang Zeng, Changjun Deng, Peixing Wang, Liyi Fan, Baodong Zheng, Yi Zhang

**Affiliations:** ^1^ China‐Ireland International Cooperation Centre for Food Material Science and Structure Design Fujian Agriculture and Forestry University Fuzhou China; ^2^ College of Food Science Fujian Agriculture and Forestry University Fuzhou China

**Keywords:** chips, oil oxidation, silver carp, surimi, vacuum frying

## Abstract

In this study, we explored the feasibility of vacuum frying to produce crisp silver carp surimi chips. The influence of three process parameters (frying temperature, frying time, and slice thickness) on the quality parameters of vacuum‐fried surimi chips (oil uptake, crispness, and optical properties) was investigated. The experimental results showed the optimal conditions were chosen as 2‐mm surimi slice being vacuum‐fried at 118°C for 2.5 min. Under these conditions, the oil content, breaking force, and color difference to commercial potato chips were 24.33%, 15.21 N, and 14.03, respectively. Additionally, we also measured the water loss during vacuum frying and the oil quality changes during storage of surimi chips. Results demonstrated the rapid loss of water content of surimi chips during vacuum frying and oil deterioration was kept at acceptable low level up to 100 days. Taken together, our study supported the applicability of vacuum frying technology to produce high‐quality silver carp surimi chips.

## 1 INTRODUCTION

Surimi, a Japanese term literally meaning “minced meat” derived from fish, is a traditional seafood and primary ingredient for making surimi‐based foods consumed worldwide (Pigott, 1986). The industrialized surimi production originated in Japan in the late 1960s, which was considered as an innovation to revitalize fish industry and make better use of “low‐value fish” (Kelsky, 1990). Currently, around 3 million tons of fish is used for the production of surimi or surimi‐based products annually (Pangsorn, Laongmanee, & Siriraksophon, 2007). The most commonly used fish for surimi production includes the threadfin bream (*Nemipterus* spp.), bigeye snapper (*Priacanthus* spp.), croaker (*Pennahia* and *Johnius* spp.), and lizardfish (*Saurida* spp.) (Benjakul, Chantarasuwan, & Visessanguan, 2003). The United States, Japan, Thailand, and China are the leading countries for the production of surimi (Vidalgiraud & Chateau, 2007).

Up to date, surimi‐based products mainly include fish balls, crabsticks, fish cake, chikuwa, and narutomaki (Park, Lin, & Yongsawatdigul, 1997). However, these traditional surimi‐based products are highly susceptible to microbial spoilage and hence need cold chain to preserve. Drying is one of the most common food preservation methods (Velescu, Tenu, Carlescu, & Dobre, 2013). Therefore, surimi chips are a promising format of surimi‐based snack to be developed as an inexpensive, tasty, and easily available snack. In addition, since chips might be one of the most popular snacks (Huang & Zhang, 2012), the development of surimi chips may also make the surimi‐based foods more marketable.

Currently, a number of novel drying techniques (such as hot air drying, vacuum drying, freeze drying, and microwave frying) have been developed for the food industry besides the conventional atmospheric frying (Qing‐guo, Min, Mujumdar, Wei‐hua, & Jin‐cai, 2006). Among these novel drying methods, the vacuum frying process is a promising technique for healthier fried foods with many advantages including lowering the oil absorption; keeping natural color and flavor; preserving more vitamins and minerals; and decreasing the formation of carcinogenic toxins (e.g., acrylamide and furan) (Dueik & Bouchon, 2011; Moreira, 2014). Indeed, this technology has been reported to successfully produce vacuum‐fried chips of potatoes, banana, carrots, apples, and kiwifruit (Belkova et al., 2018; Diamante, Savage, & Vanhanen, 2012; Shyu, Hau, & Hwang, 2005; Shyu & Hwang, 2001; Sothornvit, 2011).

In this study, we explored the feasibility of using the silver carp (*Hypophthalmichthys molitrix*) surimi as ingredient to produce crisp surimi chips by vacuum frying. The effects of three process parameters (including temperature, frying time, and slice thickness) on the oil uptake, crispness, and optical properties of vacuum‐fried surimi chips were investigated. Additionally, we also measured the water loss during vacuum frying and the oxidation of the fatty acids during storage of surimi chips. Taken together, the results supported the use of the vacuum frying technology as a method for producing high‐quality surimi chips.

## 2 MATERIALS AND METHODS

### 2.1 Materials and chemicals

Vacuum‐packaged frozen *Nemipterus virgatus* surimi was provided by Haixin Foods Co., Ltd, and stored in freezer (−30°C). Surimi was thawed prior to the experiments. Palm oil and sucrose fatty acid esters were also supplied by Haixin Food Co., Ltd. Sodium chloride was purchased from China National Salt Industry Corporation. Cassava starch and soy protein isolates were purchased from Angel Yeast Co., Ltd.

### 2.2 Preparation of surimi samples

Surimi was chopped at 1,800 rpm for 2 min using a silent cutter machine (JYL‐D020, Joyoung Co., Ltd) before sucrose fatty acid esters, sodium chloride, cassava starch, soy protein isolates, and ice were added (as shown in Table S1) and chopping continued for another 2 min. After chopping, the sample was steamed at 95°C for 5 min and frozen again before sliced into slices of 1–5 mm using a meat slicer (300ST‐12, Itop Kitchen Equipment Co., Ltd).

### 2.3 Vacuum frying

Vacuum frying experiments were performed in a vacuum fryer (QS‐05, Quanshi Food Machinery Co., Ltd). Surimi slices were placed in the frying basket, and the lid was closed. Once the desired oil temperature and vacuum level were reached, the basket was lowered into the oil and vacuum‐fried for the required frying time before the surimi slices were lifted from the oil, and then, the oil was removed by centrifuge at 200 rpm for 2 min. Then, the vacuum‐fried surimi chips were cooled down to room temperature on paper towel prior to further analysis.

### 2.4 Oil content measurement

Vacuum‐fried surimi crisps were subjected to Soxhlet extraction by hexane to measure the oil content according to the AOAC Official Method 972.28 (AOAC, 1995) using a SOX406 Fat Analyzer (Jinan Hanon Instruments Co., Ltd.). Briefly, fried samples were grinded into the size of 20 meshes using a mortar and then extracted by diethyl ether in a Soxhlet extraction barrel with dropping bottle at 65°C. The extracts were then dried in 103 ± 2°C until constant weight and weighted after cooling to air temperature. The oil content was expressed as g oil/100 g dry sample.

### 2.5 Analysis of textural properties

Texture profile analysis (TPA) of the vacuum‐fried surimi chips was carried out using TA.XT2i texture analyzer (Stable Micro Systems) equipped with a cylindrical probe P/50. The force was applied to the sample by using P/50 probe until the sample was cracked. The pretest speed, test speed and post‐test speed were set at 2.0, 1.0, 10.0 mm/s. The distance was 3 mm. The trigger type was 'auto'. The deformation ration in the tests was set as 30%, the interval stopping time was 5 s, and the trigger force was 5 g. The force–time curve was recorded and analyzed using Texture Exponent 32 software (Stable Micro Systems). The maximum breaking force was recorded to reflect the crispness of surimi chips as previous report (Diamante et al., 2012).

### 2.6 Color measurement

Color of surimi chips was measured in terms of Hunter parameters (lightness [*L* ^*^], greenness [*a* ^*^], and yellowness [*b* ^*^]) using colorimeter (ADCI‐60‐C, Beijing CTK Co., Ltd), which were thereafter compared to lightness ($L_{\text{c}}^{*}$), greenness ($a_{\text{c}}^{*}$), and yellowness ($b_{\text{c}}^{*}$) of commercial potato chips (LAY'S^®^ Classic Potato Chips). The color difference (Δ*E*) was calculated according to the following equation given below as previous report (Song, Zhang, & Mujumdar, 2007):

$$\text{Color difference}\;\left(\Delta E\right)={\left[{\left(L^{*}-L_{\text{c}}^{*}\right)}^{2}+{\left(a^{*}-a_{\text{c}}^{*}\right)}^{2}+{\left(b^{*}-b_{\text{c}}^{*}\right)}^{2}\right]}^{1/2}.$$

### 2.7 Single‐factor experiments

To optimize the process parameters for vacuum frying, initial single‐factor tests were conducted before response surface experiments to screen the appropriate influential ranges of variables, including temperature (95–135°C), frying time (1–5 min), and slice thickness (1–5 mm).

### 2.8 Box–Behnken design (BBD) test

According to the results of single‐factor experiments, a Box–Behnken response surface design with three independent variables was established (as shown in Table 1). Three independent variables were coded at three levels (−1, 0, and 1) on three responses (including oil content, breaking force, and chromatic aberration), which resulted in an experimental design of 17‐run experiments using a Design–Expert software (version 8.0.6) (Table 1). The analysis of variance (ANOVA) for the response surface quadratic model was also performed using Design–Expert software and is given in Table 2.

**Table 1:** Box–Behnken experimental design and the response for 17 experimental runs

Runs	Independent variables			Responses		
	Frying temp (°C)	Frying time (min)	Slick thickness (mm)	Oil content (%)	Δ*E*	Breaking force (N)
1	0	−1.00	−1	35.7602	18.6881	15.163
2	−1	0.00	1	26.1433	17.9548	15.916
3	−1	0.00	−1	31.2633	20.1633	15.685
4	1	0.00	−1	33.3133	17.9366	14.742
5	1	0.00	1	30.237	13.6854	15.541
6	−1	−1.00	0	28.4764	19.1534	15.664
7	0	0.00	0	23.6367	14.0703	15.461
8	0	0.00	0	22.5367	13.3203	15.321
9	1	−1.00	0	31.1067	15.0189	15.241
10	−1	1.00	0	28.6933	18.9242	16.363
11	0	1.00	−1	32.0867	16.756	15.247
12	0	−1.00	1	24.6433	15.2853	15.341
13	0	1.00	1	29.9867	14.3255	16.298
14	0	0.00	0	24.2367	14.7703	15.511
15	0	0.00	0	23.3367	13.7703	15.461
16	1	1.00	0	34.542	13.8964	15.359
17	0	0.00	0	23.1367	14.0703	15.456

**Table 2:** Regression equations (for the coded variables) and ANOVA for fitted models

Source	SS	*df*	MS	*F*‐value	*p*‐value	*R* ^2^	Adeq. Precision
Oil content (%)							
Model	292.18	9	32.46	38.55	<0.0001	0.9802	16.350
Residual	5.90	7	0.84				
Lack of fit	4.32	3	1.44	3.67	0.1208		
Pure error	1.57	4	0.39				
Corrected total	298.08	16					
Regression equations: R_1_ = 23.38 + 1.83*A* + 0.67*B*−2.68*C* + 0.80*AB* + 0.51*AC* + 2.25*BC* + 3.47*A* ^2^ + 3.85*B* ^2^ + 3.39*C* ^2^							
Δ*E*							
Model	84.01	9	9.33	27.78	0.0001	0.9728	15.718
Residual	2.35	7	0.34				
Lack of fit	1.23	3	0.41	1.47	0.3490		
Pure error	1.12	4	0.28				
Corrected total	86.37	16					
Regression equations: R_2_ = 14.00–1.96*A*–0.53*B*–1.54*C*–0.22*AB*–0.51*AC* + 0.24*BC* + 1.96*A* ^2^ + 0.79*B* ^2^ + 1.48*C* ^2^							
Breaking force (N)							
Model	2.48	9	0.28	58.44	<0.0001	0.9869	31.773
Residual	0.033	7	4.718E‐003				
Lack of fit	0.013	3	4.236E‐003	0.83	0.5411		
Pure error	0.020	4	5.080E‐003				
Corrected total	2.51	16					
Regression equations: R_3_ = 15.44–0.34*A* + 0.23*B* + 0.28*C*–0.15*AB* + 0.14*AC* + 0.22*BC* + 0.087*A* ^2^ + 0.13*B* ^2^–0.058*C* ^2^							

Note

R_1_: oil content (%); R_2_: Δ*E*; R_3_: breaking force; *A*: frying temperature; *B*: frying time; and *C*: slick thickness; *A*, *B*, and *C* were the coded variables for −1 to 1 according to Table 1. Regression terms in bold were significant (*p* < 0.05).

Abbreviations: MS, mean square; SS, sum of squares.

### 2.9 Moisture content measurement

The water content of surimi chips was measured by the oven‐drying method (AOAC Official Method 984.25) (Shiroma & Rodriguez‐Saona, 2009). The water content was expressed as g water/100 g total.

### 2.10 Determination of acid values (AVs) and peroxide values (POVs)

The acid value (AV) and peroxide value (POV) of the oils were determined using a titration method according to the AOAC Official Method 940.28 and AOAC Official Method 965.33, respectively (Mostaghimi, 2015; Quiroz‐Moreno, Fontes‐Gagiola, Rouzaud‐Sández, & Vidal‐Quintanar, 2013).

### 2.11 Statistical analysis

All data were presented as means ± standard deviation. As for multiple group comparison, the significance of the differences among the treatment groups and their respective control groups was analyzed using Origin 8.0 software. Statistical significance was assessed by either Student's *t* test or one‐way analysis of variance (ANOVA) followed by Tukey's multiple comparison. Differences between means were considered statistically significant if *p* < 0.05. All the experiments were carried out at least in three independent experiments.

## 3 RESULTS

### 3.1 Single‐factor experiments for optimization of vacuum frying conditions

**Figure 1:**
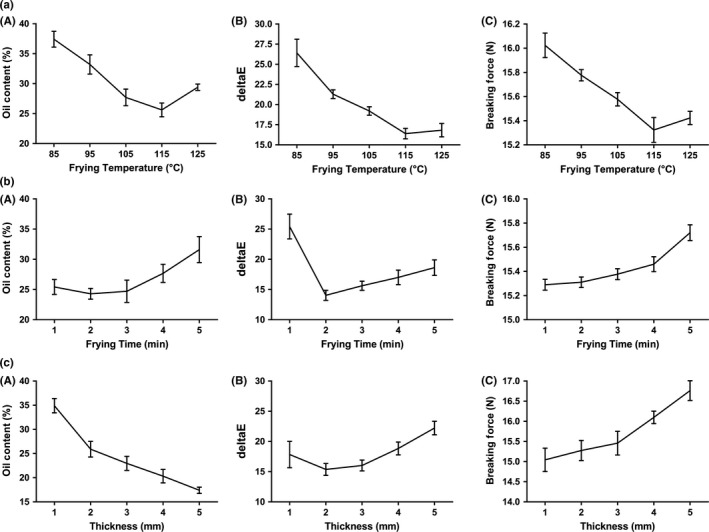
Effects of frying temperature, frying time, and slice thickness on the oil content, crispness, and color difference to commercial potato chips. All the experiments were carried out by one factor being changed ([a] frying temperature; [b] frying time; and [c] slice thickness), while the other two factors were set constant (frying temperature: 115; frying time: 3 min; or slice thickness: 3 mm). Values are presented as mean ± *SD* with three replicates

We first determined the effects of frying temperature on the quality characteristics of surimi chips. As shown in Figure 1A, the oil content of surimi chips rapidly decreased with the increase in temperature from 85 to 105°C and then kept at low levels when temperature was above 105°C. This decreasing trend of oil content with frying temperature increasing may be due to lower oil viscosity at the higher temperatures which may facilitate oil drainage (Esan, Sobukola, Sanni, Bakare, & Munoz, 2015; Yagua & Moreira, 2011). Regarding the breaking force and color difference to commercial potato chips (Δ*E*), a similar trend was observed for both that higher temperature resulted in decreased breaking force and Δ*E* from 85 to 115°C and then slightly increased at 125°C. Since our goal is to achieve low oil content, low breaking force, and similar color to commercial chips (low Δ*E*), 115°C was selected as the central point for following response surface optimization.

Figure 1B demonstrates the effect of frying time on the quality characteristics of surimi chips. The oil content was kept at low level when frying time was from 1 to 3 min, then increased gradually along the extended frying time more than 3 min. This was also observed in previous studies that extended frying time is associated with higher oil content (Krokida, Oreopoulou, & Maroulis, 2000; Zhang, Zhang, Fan, Li, & Fan, 2018). Similarly, the Δ*E* dramatically fell in the first 2 min but showed upward trend after 2 min of frying, while the breaking force kept at low level in the first 4 min and rapidly increased with the increasing frying time more than 4 min. This may be due to the reason that in the initial stage of vacuum frying, the internal moisture quickly evaporated to create air pores on the surface, while in the secondary stage, the samples became harder by dehydration with higher oil uptake (van Koerten, Schutyser, Somsen, & Boom, 2015a, 2015b). Thus, 3 min of frying time was selected as the optimized concentration in the following response surface methodology research.

The effects of slice thickness were next to be explored. As seen in Figure 1C, the oil content gradually decreased, while the breaking force showed a general upward trend by increasing slice thickness up to 5 mm. In several previous studies investigating the effects of slice thickness on final oil content of potato crisps, a similar trend was also observed that increasing the potato crisps thickness decreased the final oil content (Krokida et al., 2000). Meanwhile, as expected, the breaking force increased with the increase in slice thickness, which is similar to previous findings reported by George O. Abong et al. (Abong, Okoth, Imungi, & Kabira, 2011). In addition, the Δ*E* declined to lowest point at 2 mm and then increased. Therefore, the central point of slice thickness for the following response surface optimization was set as 2 mm.

### 3.2 Response surface methodology for optimization of vacuum frying conditions

**Figure 2:**
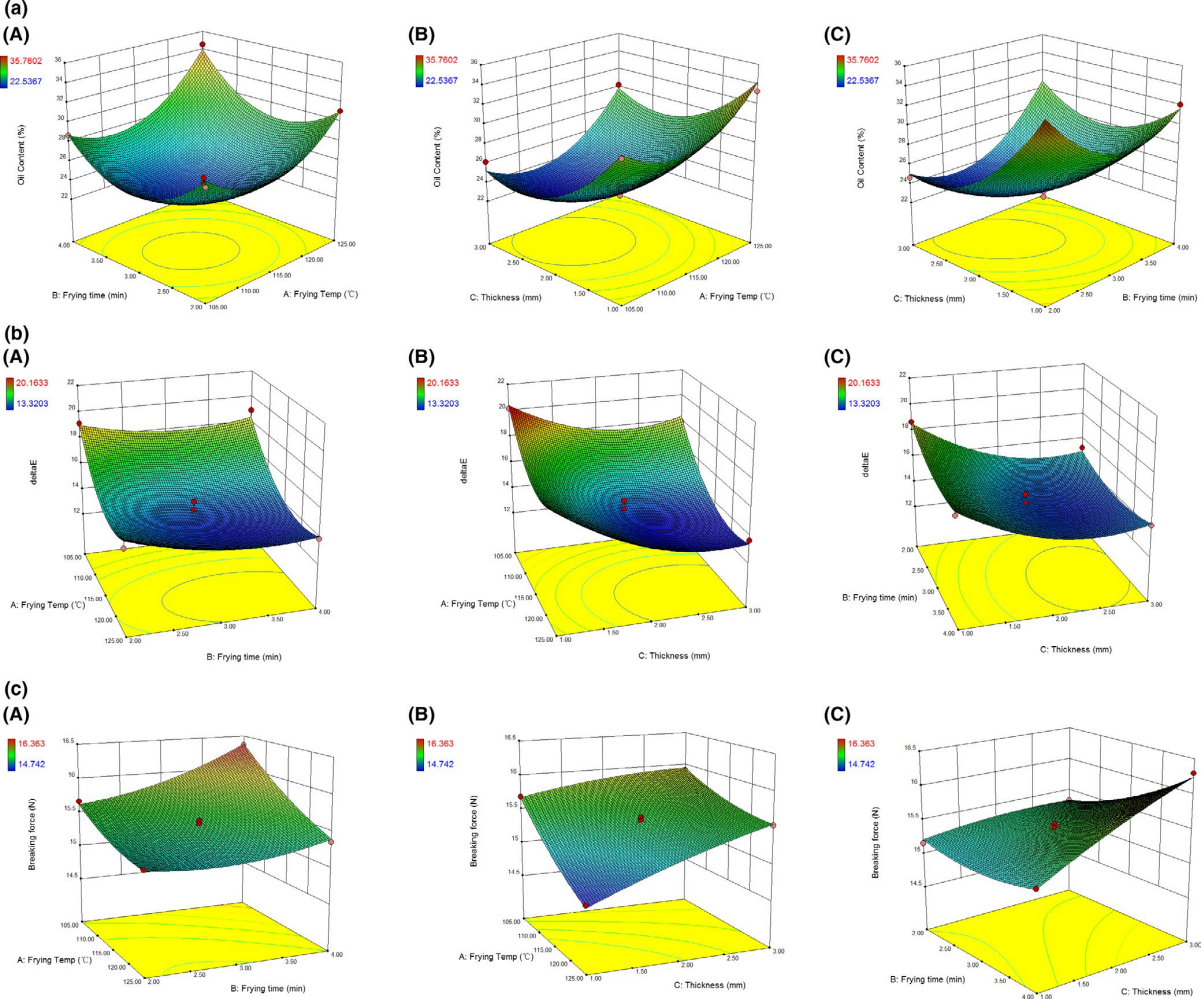
Response surface 3D surface plots and contour plots for oil content (a), crispness (b), and color difference to commercial potato chips (c) of vacuum‐fried surimi chips, as affected by frying temperature and frying time (A); frying temperature and slice thickness (B); or frying time and slice thickness (C) when the third factor is set at level 0

Since the frying is a process involving complex heat transfer and chemical reactions (Gertz, 2014), to reflect the possible interaction effects arising between factors, response surface methodology was also employed in this study. The design matrix of response surface methodology experiments is shown in Table 1. Table 1 also presents the experimental responses for all the experimental runs, while response surface 3D surface plots and contour plots for these experimental responses are illustrated in Figure 2.

By multiple regression analysis, regression equations and statistical parameters for the experimental responses were obtained as shown in Tables 2 and 3. The analysis of variance (ANOVA) indicated all the regression models reached a significant level (*p* < 0.05) with satisfactory determination coefficients (*R* ^2^), while all the lack‐of‐fit values were more than 0.05, indicating that the lack‐of‐fit values were not significant relative to the pure error.

**Table 3:** Regression coefficient table for different response using coded factors

Term	R_1_			R_2_			R_3_		
	Coefficient	*F*‐value	*p*‐value	Coefficient	*F*‐value	*p*‐value	Coefficient	*F*‐value	*p*‐value
Intercept	23.38			14.00			15.44		
*A*	1.83	31.73	0.0008	−1.96	91.21	<0.0001	−0.34	199.63	<0.0001
*B*	0.67	4.20	0.0795	−0.53	6.70	0.0360	0.23	91.46	<0.0001
*C*	−2.68	68.05	<0.0001	−1.54	56.22	0.0001	0.28	135.20	<0.0001
*AB*	0.80	3.07	0.1230	−0.22	0.59	0.4662	−0.15	17.89	0.0039
*AC*	0.51	1.24	0.3023	−0.51	3.10	0.1215	0.14	17.09	0.0044
*BC*	2.25	24.13	0.0017	0.24	0.70	0.4294	0.22	40.38	0.0004
*A* ^2^	3.47	60.33	0.0001	1.96	48.12	0.0002	0.087	6.72	0.0359
*B* ^2^	3.85	74.25	<0.0001	0.79	7.79	0.0269	0.13	14.62	0.0065
*C* ^2^	3.39	57.40	0.0001	1.48	27.27	0.0012	−0.058	2.98	0.1281

Response surface 3D surface plots and contour plots are shown in Figure 2 to illustrate the interactions of the variables. Each figure shows the effects of two factors on the responses, while the other one was set at level 0. As shown in Figure 2A, where the oil content of surimi chips was given as a function of frying temperature and frying time at constant slice thickness, the oil content decreased with the increase in frying time from 2 to ~2.75 min. But beyond 2.75 min, the oil content decreased with increasing frying time (Figure 2A‐a). Similarly, when the frying time was set, the oil content was found to decrease with increase in frying temperature from 105 to ~112°C; then, it showed rapid uptrend when the frying temperature continued to increase. Figure 2A‐b shows the 3D surface plot and contour plot at varying frying temperature and slice thickness at fixed frying time, indicating that the oil content decreased with increasing frying temperature at the initial stage and then rapidly increased, while increasing slice thickness from 1 mm to around 2.5 mm lowered the oil content but further increasing of thickness would slightly increase the oil content. Figure 2A‐c indicates the relationship between the oil content and two variables of frying time and thickness when frying temperature was set as level 0. As far as the figure is concerned, the oil content increased when the frying time extended from 2 to ~2.75 min and then began to decrease. At a fixed frying time, the oil content declined with increasing thickness from 1.0 mm to ~2.25 mm, but beyond 2.25 mm, oil content slightly increased again with increasing thickness.

Breaking force values from 14.742 to 16.363 N have been obtained. From the 3D surface plots and contour plots shown in Figure 2B, it could be observed that in the whole range of frying temperature and frying time being tested, these two factors had positive effects on the breaking force of surimi chips, while the breaking force was enhanced with increasing thickness at the initial stage but further increasing of thickness would decrease the breaking force.

The color difference to commercial potato chips (Δ*E*) ranged from 13.3203 to 20.1633 was recorded in our tests. As shown in Figure 2C, frying temperature and slice thickness greatly affected the Δ*E*, which was decreased by increasing the frying temperature and slice thickness. In contrast, only a little change in the Δ*E* was observed when frying time extended from 2 to 4 min. This was also reflected by ANOVA.

Taken together, these results are in accordance with single‐factor test and the ANOVA. Based on our purpose to achieve low oil uptake, low Δ*E*, and low breaking force, the optimal condition of vacuum frying for producing surimi chips was next determined by Design–Expert software, which was chosen as 2.24‐mm surimi slice being vacuum‐fried at 117.85°C for 2.70 min. Upon this condition, the theoretical oil uptake, breaking force, and color difference to commercial potato chips (Δ*E*) were predicted to be 23.67%, 15.37 N, and 13.51, respectively. The suitability of the models to predict the responses was next tested under this selected optimum condition. As shown in Table 4, considering the actual operation convenience, the optimal conditions for adjusting the condition were chosen as 2‐mm surimi slice being vacuum‐fried at 118°C for 2.5 min. The experimental results were found to be in agreement with the theoretical responses, suggesting the obtained model in this study was accurate and adequate to guide the production of vacuum‐fried surimi chips.

**Table 4:** Predicted and experimental response values at optimum conditions

Trials	Frying temp	Frying time (min)	Slice thickness (mm)	Oil content (%)	Δ*E*	Breaking force (N)
Predicted	117.85	2.70	2.24	23.67	13.51	15.37
Experimental	118	2.5	2	24.33 ± 0.69^a^	14.53 ± 1.01^a^	15.21 ± 1.19^a^

Note

a Mean ± standard deviation for three replicates.

### 3.3 Determination of the water loss during vacuum frying

**Figure 3:**
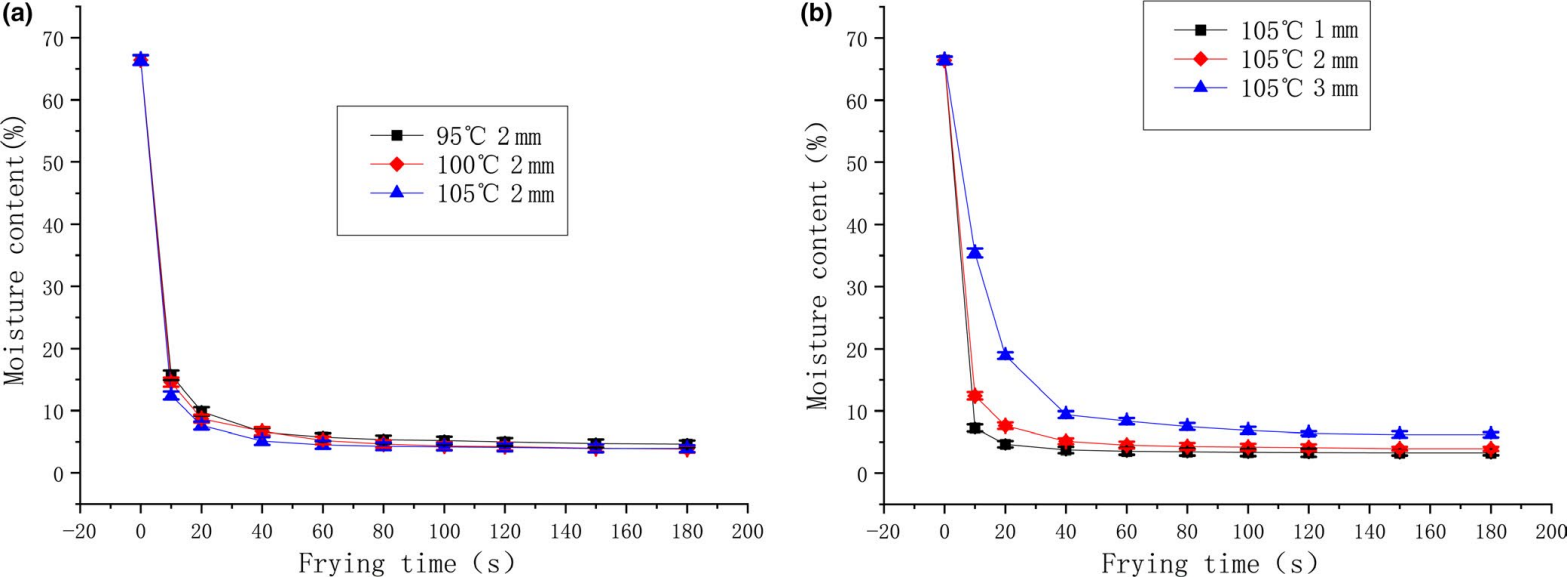
The moisture content of vacuum‐fried surimi chips during vacuum frying, as affected by frying temperature and time (a); and slice thickness and frying time (b)

Next, we evaluated the water loss of surimi chips during vacuum frying. As shown in Figure 3, there is a rapid water loss in the initial stage of vacuum frying before leveling off after around 25–40 s. This phenomenon was also revealed by previous studies (Krokida et al., 2000), which may be due to the depletion of nearly all free water and the formation of crust to stop the water evaporation. Moreover, results also revealed that decreasing the slick thickness accelerated the water loss, while increasing the frying temperature (in the range of 95–105°C) had little positive influence on the water loss.

### 3.4 Determination of the acid value and peroxide value of surimi chips during storage

**Figure 4:**
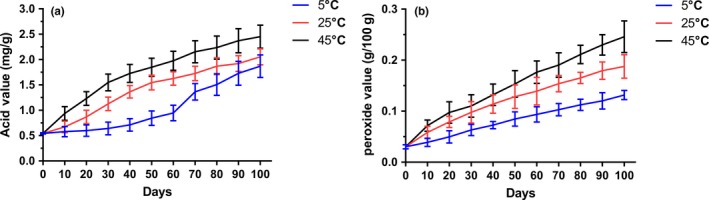
The acid value (a) and peroxide value (b) of surimi chips during storage up to 100 days. Nitrogen‐filled packaged vacuum‐fried surimi chips were stored at 5°C (blue line), 25°C (red line), and 45°C (black line) to test the oxidative stability of lipid

Lipid oxidation during storage is one of the major factors causing the deterioration of the sensorial proprieties of chips. Therefore, to explore the oil quality stability of surimi chips produced by vacuum frying, we also measured the changes in acid value and peroxide value of surimi chips during the storage. Here, we adopt nitrogen‐filled packaging, a common method used for commercial chips package to store fried surimi chips. As shown in Figure 4, both the acid value (Figure 4A) and peroxide value (Figure 4B) of nitrogen‐filled packaged silver carp surimi chips were kept at low level for nearly 100 days. According to the regulations of frying fats and oils widely adopted in various nations (e.g., the limitation of acid value was as 2 [e.g., in Finland] ‐ 4.5 [e.g., in the Netherlands]) (Paul & Mittal, 1997), the results indicated that nitrogen‐filled packaging is a suitable method for preventing lipid oxidation of vacuum‐fried surimi chips during its storage.

## 4 DISCUSSION

The global market for healthy snacks is steadily growing, enhanced by the increasing demand of consumers for convenience products with high nutritive and sensory qualities (Sun‐Waterhouse, 2011). As one of the most favorable snacks, chips occupy a very significant chunk of the snack market (Brennan, Derbyshire, Tiwari, & Brennan, 2013). Meanwhile, surimi is a useful ingredient for producing various kinds of processed foods (Pigott, 1986). According to the United States Department of Agriculture National Nutrient Database, fish surimi contains around 76% water, 15% protein, 6.85% carbohydrate, 0.9% fat, and 0.03% cholesterol (Yousefi & Moosavi‐Nasab, 2014). However, to the best of our knowledge, producing surimi chips has not been reported. Therefore, development of new surimi‐based snack products (such as surimi chips) is needed. Here, we explored the vacuum frying as a useful technique for making nutritional snack of crispy surimi chips with minimum oil content.

Vacuum frying is a frying technology that is performed under the pressure below atmosphere levels (Pandey & Moreira, 2012). Utilization of vacuum frying instead of conventional atmospheric frying has been proven to provide better heat and mass transfer rates. This method has been reported to be successfully applied to make fruits chips and preserve their natural flavors and aromas, and result in a good crispy texture.

In particular, previous studies have pointed out that vacuum frying appears to be a suitable method for chips production due to its low temperature and low oxygen level when operated in a closed system, which can significantly lower the boiling point of water and lead to the products with low oil content, desired sensory and nutritional quality, and less undesirable carcinogenic toxins (Dueik & Bouchon, 2011). Here, our results also demonstrated vacuum frying of surimi slices is a promising process enabling production of surimi‐based crisps with low oil content at 24.33% at optimal condition.

In addition, the results of the experiment also showed that color of vacuum‐fried surimi chips was comparable to control commercial potato chips, which may make the surimi chips more acceptable to consumers. Meanwhile, the water loss curve also supported the dehydration process during the vacuum frying was quickly achieved, which might contribute to the decrease in energy consumption (Maity, Bawa, & Raju, 2014). Indeed, previous studies have suggested that the moisture loss during the vacuum frying determines the oil absorption *via* affecting the extent of crust formation and the volume that is available for oil infiltration (Dueik, Robert, & Bouchon, 2010; Shyu et al., 2005). Besides, we also determined the oxidative stability of lipid of vacuum‐fried surimi chips. The results indicated that using nitrogen‐filled packaging to store vacuum‐fried surimi chips could keep both the acid value and peroxide value at acceptable low level up to 100 days, showing its stability might not be an obstacle to developing commercial surimi chips.

Admittedly, the present study was conducted with small sample size, and thus, before final recommendation of this technology for its industrial use, confirmations are required by using increased sample sizes.

## 5 CONCLUSION

In conclusion, our study here supported that the vacuum frying technique could be utilized for producing high‐quality fried surimi‐based chips with low oil content and similar color to commercial potato chips. Frying temperature, frying time, and slice thickness demonstrated significant effects on the quality attributes of vacuum‐fried surimi chips.

## CONFLICT OF INTEREST

The authors declare no conflict of interest.

## ETHICAL STATEMENT

This study does not involve any human nor animal testing.
