# Supplementary material for: Optimization of vacuum frying condition for producing silver carp surimi chips
Source: Food Sci Nutr. 2019 Jul 3;7(8):2517–26. doi: 10.1002/fsn3.1077 (PMC6694419; doi:10.1002/fsn3.1077)
Supplement: Supplementary file 1 [file FSN3-7-2517-s001.docx]

**Supplementary Table 1** Formulation of surimi paste used for producing surimi chips

| Ingredient | Weight (g) |
| --- | --- |
| Silver CarpSurimi | 100 |
| Cassava starch | 20 |
| Soy protein isolates | 20 |
| Sucrose fatty acid esters | 15 |
| Sodium chloride | 2 |
| Ice | 100 |
